# Percutaneous endoscopic-assisted direct repair of pars defect without general anesthesia could be a satisfying treatment alternative for young patient with symptomatic lumbar spondylolysis: a technique note with case series

**DOI:** 10.1186/s12891-020-03365-4

**Published:** 2020-06-02

**Authors:** Mengran Jin, Jun Zhang, Haiyu Shao, Jianwen Liu, Tingxiao Zhao, Yazeng Huang

**Affiliations:** 1 Department of Orthopaedics, Zhejiang Provincial People’s Hospital, Shangtang Road No. 158, Hangzhou, 310014 Zhejiang Province China; 2 People’s Hospital of Hangzhou Medical College, Zhejiang Provincial People’s Hospital, Hangzhou, 310014 Zhejiang Province China

**Keywords:** Endoscopic, Direct repair, Lumbar spondylolysis, Pars defect, Minimally invasive surgery

## Abstract

**Background:**

Multiple surgical procedures are applied in young patients with symptomatic lumbar spondylolysis when conservative treatments fail. Although the optimal surgical procedure option is controversial, the treatment paradigm has shifted from open surgery to minimally invasive spine surgery. To date, a limited number of studies on the feasibility of percutaneous endoscopic-assisted direct repair of pars defect have been carried out. Herein, for the first time, we retrospectively explore the outcomes of pars defect via percutaneous endoscopy.

**Methods:**

We retrospectively examined young patients with spondylolysis treated using the percutaneous endoscopic-assisted direct repair of pars defect supplemented with autograft as well as percutaneous pedicle screw fixation between September 2014 and December 2018. Six patients with a mean age of 18.8 years were enrolled in the study. We used preoperatively computed tomographic (CT) scans to evaluate the size of pars defect, and graded disc degeneration using Pfirrmann’s classification through magnetic resonance images (MRI). We assessed the clinical outcomes using the Oswestry Disability Index (ODI), 36-Item Short-Form Health Survey (SF-36) as well as Visual Analogue Scale for back pain (VAS-B).

**Results:**

Our findings revealed that pain intensity and function outcomes, including VAS-B, ODI, and SF-36 (PCS and MCS) scores, were markedly improved after surgery and at the final follow-up visit. The change in the gap distance of the pars defect was remarkably significant after surgery and during the follow-up period. Only one of the 12 pars repaired was reported as a non-union at the final follow-up visit. Moreover, no surgery-related complications were reported in any of the cases.

**Conclusion:**

Percutaneous endoscopic-assisted direct repair of pars defect without general anesthesia, a minimally invasive treatment option, supplemented with autograft and percutaneous pedicle screw fixation, could be a satisfying treatment alternative for young patients with symptomatic lumbar spondylolysis.

## Background

Lumbar spondylolysis, isthmic lysis, or discontinuity of the vertebral pars interarticularis, constitutes a comparatively common condition that causes low back pain (LBP) among young patients. The disease affects approximately 3–6% of the general population, and notably, 15% of athletes [1–3]. Nonsurgical managements, including physical therapy, activity modification, and bracing, remain the primary form of treatment of symptomatic lumbar spondylolysis and are successful in a considerable number of patients. Surgical intervention is usually indicated after 6 months of unsuccessful conservative management, with chronic pain, and non-union over 9–12 months. Moreover, a select group of active individuals, especially athletes, are treated with surgery due to the high demand to resume their livelihood [4].

Therefore, multiple techniques and some modifications have been reported for the surgical fixation of pars defects [3, 5–8]. Direct repair (DR) of the pars defect is more favorably used due to its inherent advantages over the other surgical methods. Theoretically, DR maintains the motion of the affected segment and refrains from the problems of the adjacent segments. However, divergent findings have been reported regarding the efficacy of DR techniques. Union rate after DR is only approximately 52% due to insufficient manipulation of the pars defect and segmental instability [9, 10]. Besides, most of the aforementioned surgical techniques are performed under general anesthesia.

In the present study, we described a retrospective analysis of collected data to analyze the clinical outcomes of six patients who underwent percutaneous endoscopic-assisted repair of the pars defect supplemented with percutaneous pedicle screw fixation of spondylolysis.

## Materials and methods

### Subjects

This is a non-commercialized retrospective study performed by a single surgeon at our institution, and the study has been reported as per the PROCESS criteria [11]. Consent was obtained from each patient and the Local Institutional Review Board approved the study. We retrieved the medical records 62 young patients who were surgically treated between September 2014 and December 2018 at our institution.

We enrolled the patients in the study using the following inclusion and exclusion criteria. The inclusion criteria were: (1) predominant clinical manifestation, including LBP, but not radicular pain. (2) Lumbar spine spondylolysis diagnosed using X-rays (anteroposterior, lateral, both oblique, and dynamic images), computed tomographic (CT) scans, and/or magnetic resonance image (MRI) that corresponded to clinical manifestations. (3) Bony callus or sclerosis detected using CT scans, persist with old injury. (4) Mild disc degeneration observed on MRI images. (5) Refractory to conservative treatments, including medication, bed rest as well as physical therapy for at least 6 months. (6) A follow-up of at least 12 months. The exclusion criteria constituted: (1) lumbar spine fractures, infection, or tumors. (2) disc degeneration over grade II according to the Pfirrmann classification [12]; (3) Fresh pars defect without bone callus. (4) A follow-up of fewer than 12 months. (5) Age of more than 30 years. Therefore, 6 patients were enrolled in the present study, with a mean age of 18.8 ± 2.7 years (range 16–23 years).

### Surgical technique and postoperative protocol

**Fig. 1:**
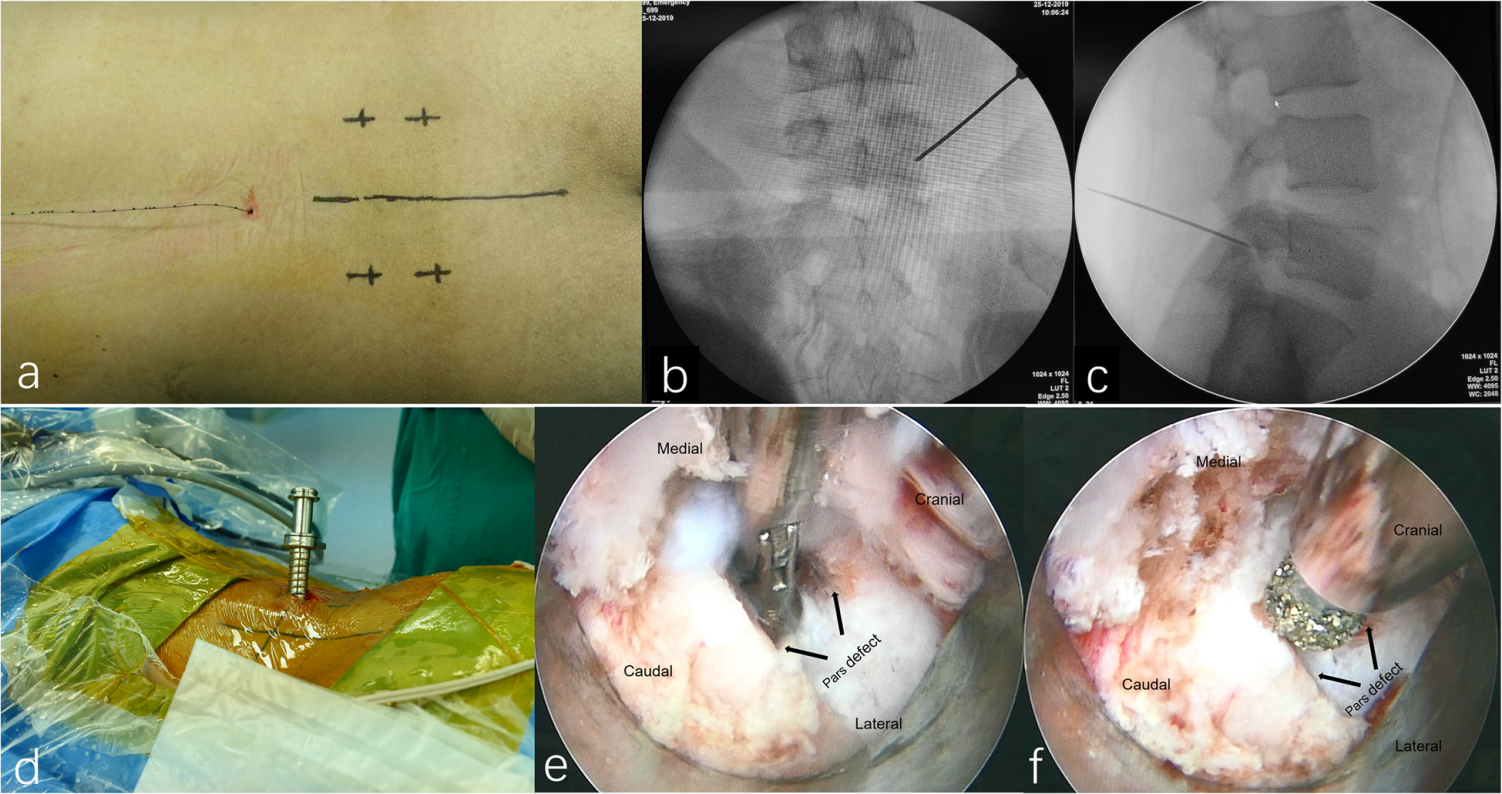
The surgery was performed under local anesthesia supplemented with epidural anesthesia (**a**); An 18-G spinal needle was inserted and navigated toward the defected pars under image intensifier (**b**, **c**); A working channel was placed properly (**d**); The scar and sclerosis were cleaned by using the forceps and bur carefully (**e, f**)

**Fig. 2:**
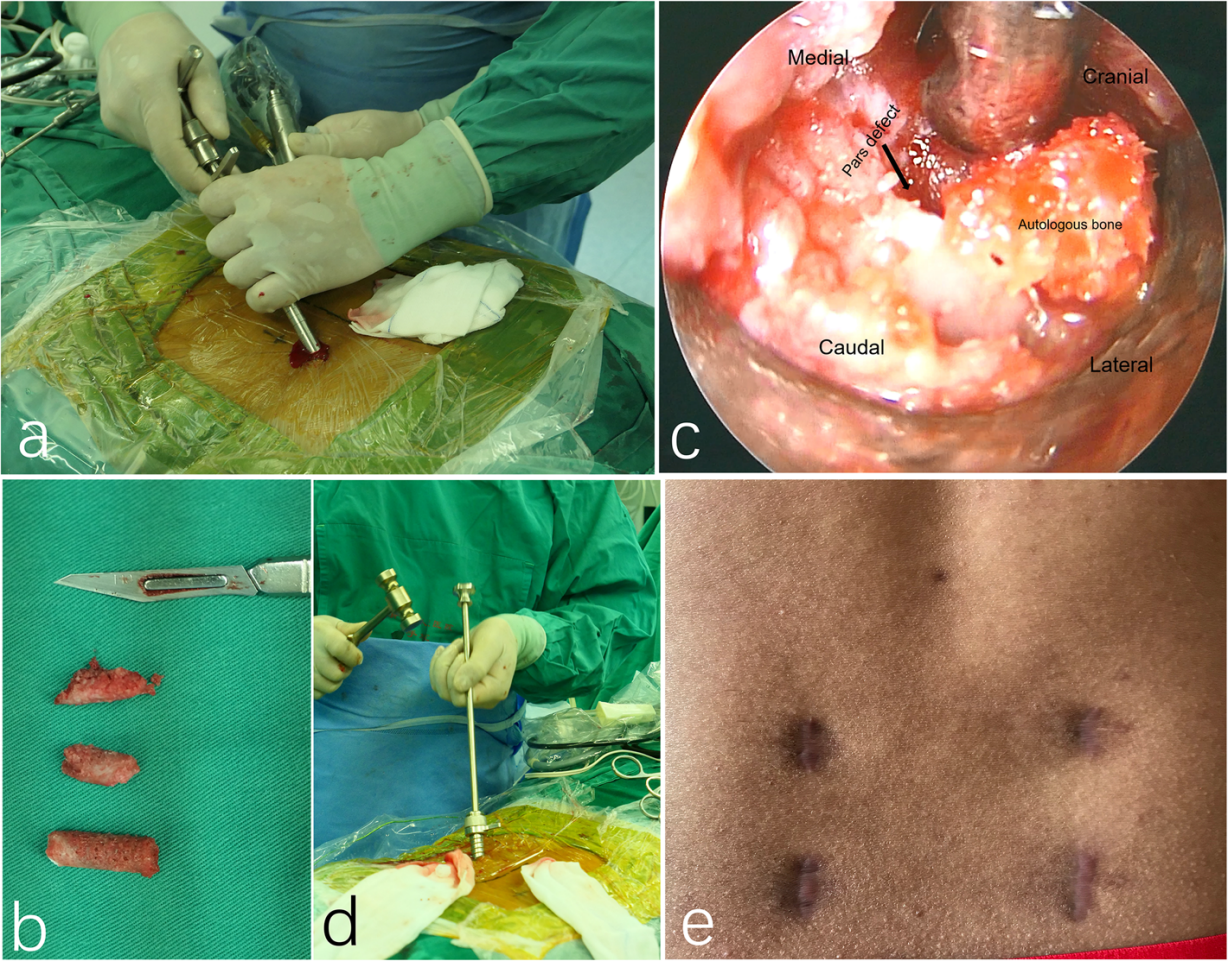
Autologous bone was harvested from the posterior superior iliac spine through the subcutaneous tunnel (**a, b**); Cancellous portion of the bone was compacted into the gap of isthmus (**c, d**); The final surgical scar of four incisions (**e**)


The surgery was performed under local anesthesia supplemented with epidural anesthesia (EA). The EA tube was placed over 2 segments above the surgical segment, usually at L2/3 (Fig. 1a). Initially, we applied 1% lidocaine 3 ml and then added 0.25% ropivacaine to adjust the sensory level and attain the aim of sensory-motor separation [13]. The patient was placed in prone position on the operating table and draped aseptically. We obtained proper anteroposterior images, depicting the parallel endplates and a centered spinous process equidistant to well-visualized pedicles. The skin incision was determined according to the CT axial images, approximately 1.5 cm long and 1 cm lateral to the lateral border of the pedicle. A combination of 1% lidocaine and 0.5% bupivacaine was administered to the skin, subcutaneous tissue and periosteum. After a 1.5 cm skin and fascia incision was made, an 18-G spinal needle was inserted and navigated toward the defected pars under image intensifier, and then replaced by a 0.8 mm blunt-tipped guidewire (Fig. 1b, c). A series of blunt obturators were introduced followed by the placement of a working channel above the obturator (Fig. 1d). We verified the final position on the anteroposterior and lateral fluoroscopic images. After introducing the endoscope, the surgery was performed. We cleaned the soft tissues constituting the para-spinal muscle to expose the pars defect using endoscopic scissors and forceps. The bleeding points were coagulated using a low-energy bipolar radiofrequency. We cleaned the scar and sclerosis using the forceps and bur carefully until blood was observed (Fig. 1e, f, supplementary video 1). Autologous bone was harvested from the posterior superior iliac spine through the subcutaneous tunnel (Fig. 2a, b, supplementary video 2) and the cancellous portion of the bone was compacted into the gap of isthmus under endoscopic visualization (Fig. 2c, d, supplementary video 3). Corticocancellous bone was compacted on the defect and the adjacent lamina area after decorticating with a bur until blood was observed. Another three 10-mm skin incisions were made after local infiltration with a combination of 1% lidocaine and 0.5% bupivacaine evenly along the tracts of percutaneous pedicle screws. Routine bilateral pedicle screw placements were attained using a commercially available percutaneous pedicle screw fixation technique (Aaxter Co., Ltd., Taiwan, China) [14]. We removed all the instruments after the final tightening of the screw extenders. Finally, all four incisions were closed using polyglycolic acid sutures (Vicryl Rapide, Ethicon, Cincinnati, OH, USA) layer by layer (Fig. 2e). We allowed all patients to ambulate on the first day following the surgery. The patients were restrained from undertaking any sports activity for 3 months. After that, patients were allowed to resume normal preoperative activities.

**Additional file 1 Supplementary video 1.**




**Additional file 2 Supplementary video 2.**




**Additional file 3 Supplementary video 3.**



### Clinical and radiographic assessment

Routine lumbar spine plain X-rays (standing anteroposterior, lateral neutral, flexion, as well as extension views), CT and MRI were obtained pre-operatively, then standing X-rays and/or CT were collected at each follow-up interval. The baseline radiological characteristics, including disc degeneration, slippage distance, and surgical level, were retrospectively evaluated. Specifically, disc degeneration was graded according to Pfirrmann’s classification [12]. The criteria used in defining bone union constituted; no radiolucency between the graft and the vertebral body, showing bridging osseous trabeculae on the CT images, and less than 3 mm of movement between the tips of the posterior spinous processes of the fused segments on flexion and extension lateral radiographs lumbar spine.

The patients were also evaluated for operation time, blood loss, duration of hospital stay, as well as the perioperative complications. We assessed clinical outcomes using the visual analogue scale for the back (VAS-B) and leg (VAS-L) pain, Oswestry disability index (ODI), and 36-Item Short Form Health Survey (SF-36) preoperatively, immediately postoperatively, and at each follow-up interval.

### Statistical analysis

We calculated the following summary statistics: means and standard deviation, and frequencies and percentages for continuous and categorical variables, respectively. Statistical verification was determined using SPSS for Windows (version 17.0.1; SPSS Inc., Chicago, IL, US). The differences in preoperative, postoperative, and final follow-up VAS, ODI, and SF-36 scores were evaluated using the paired Student t test. All significance tests were 2-tailed, with *P* < 0.05 representing statistical significance.

## Results

We enrolled 6 patients (2 males and 4 females), with a mean age of 18.8 ± 2.7 years (range 16–23 years) in this study (Table 1). The mean period of non-operative treatment before index surgery was 11.7 ± 5.5 months (range 7–22 months). L5 bilateral par defect occurred in all cases. The mean of the operation time was 113.0 ± 25.2 min (range 98–130 min), and the mean duration of hospital stay was 3.4 ± 0.8 days (range 3–4.5 days).

**Table 1:** The demographic data of all patients

Patient No.	Sex	Level	Duration of Nonoperative Treatment (mo)	Operation Time (mins)	Length of hospital stay (Day)
1	M	L5 bilateral	9	105	3
2	F	L5 bilateral	22	130	3
3	F	L5 bilateral	7	107	3
4	M	L5 bilateral	11	112	4
5	F	L5 bilateral	13	126	4.5
6	F	L5 bilateral	8	98	3

### Primary end point (pain intensity)

The VAS score for back pain was 6.9 ± 2.1 at enrollment, 2.3 ± 1.3 before discharge, and 1.1 ± 0.9 at the final follow-up, respectively. The LBP levels before discharge and at the final follow-up were markedly lower compared with the preoperative level (both *P*<0.05).

### Secondary end points

We evaluated the functional outcomes using the ODI score and SF-36 (MCS and PCS). The ODI scores were 25.7 ± 4.9% at the baseline, 13.5 ± 5.2% before discharge, and 9.8 ± 3.7% at the final follow-up, respectively. Patients indicated remarkable improvement in ODI scores from baseline to those before discharge, and at final follow-up (both *P*<0.05). The patients improved remarkably before discharge and at the final follow-up both on PCS and MCS scores, relative to the baseline scores (both *P*<0.05). The mean PCS scores improved from 36.1 ± 4.9 at admission to 48.7 ± 6.1 after surgery and 54.5 ± 4.9 at the final follow-up. The mean MCS scores improved from 42.7 ± 5.3 at enrollment to 54.3 ± 8.2 after treatment and 57.3 ± 6.8 at the final follow-up (Table 2).

**Table 2:** The radiographic and functional outcomes of all patients before surgery, after surgery and at final follow-up interval

Parameters	Gap distance (mm)	VAS-B	ODI	SF-36 (PCS)	SF-36 (MCS)
At-enrollment	3.3 ± 1.7	6.9 ± 2.1	25.7 ± 4.9	36.1 ± 4.9	42.7 ± 5.3
After surgery	2.6 ± 1.3^*^	2.3 ± 1.3^*^	13.5 ± 5.2^*^	48.7 ± 6.1^*^	54.3 ± 8.2^*^
Final follow-up	1.9 ± 1.4^†^	1.1 ± 0.9^†^	9.8 ± 3.7^†^	54.5 ± 4.9^†^	57.3 ± 6.8^†^

^*^ The postoperative values were compared with ones at enrollment, and the result were statistically significant (*P*<0.05)

^**†**^ The follow-up values were compared with ones at enrollment, and the result were statistically significant (*P*<0.05)

VAS-B: Visual Analogue Scale for Back pain; ODI: Oswestry Disability Index; SF-36: Short Form-36 Health Surgery Questionnaire; PCS: Physical Component Score; MCS, Mental Component Score

**Fig. 3:**
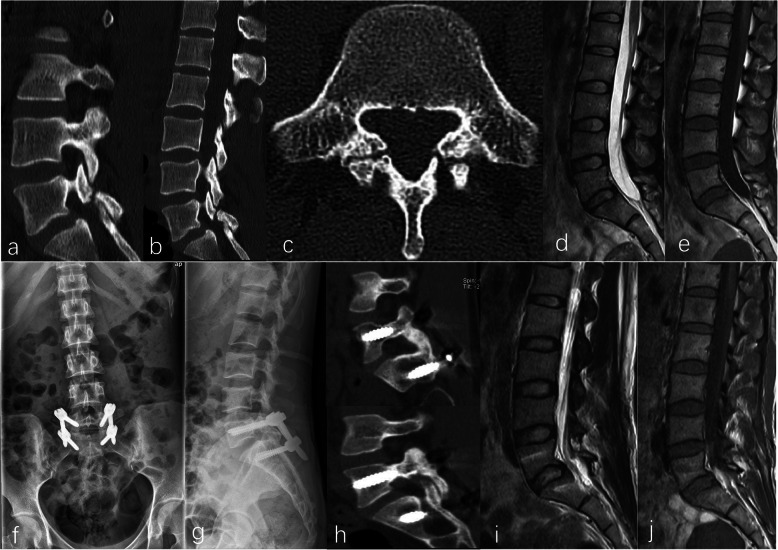
A 23-year-old woman was admitted to our institution due to chronic low back pain without lower extremity pain and numbness for 8 months. Bilateral pars defects at L5 was revealed on preoperative CT scans (**a, b, c**). Grade I disc degeneration at L5/S1 was seen on preoperative MRI images (**d, e**). The patient underwent single-level endoscopic-assisted direct repair of pars defect supplemented with percutaneous pedicle screw fixation successfully (**f, g**). Sagittal CT images at 11 months postoperatively revealed solid fusion at both sides (**h**). Obvious disc degeneration was not observed on MRI images (**i, j**)

The mean gap distance was 3.3 ± 1.7 mm at enrollment, and improved to 2.6 ± 1.3 mm before discharge, and 1.9 ± 1.4 mm at the last follow-up, respectively. The changes in the gap distance were markedly significant (*P*<0.05). The union rate at the last follow-up was 91.7% (11/12) (Fig. 3).

No case was alternated into open surgery intraoperatively, and no surgery-related perioperative complications were encountered in the present case series.

## Discussion

Lumbar spondylolysis is among the crucial causes of symptomatic low back pain and impacts an estimated 15–47% of young people [2]. According to Beutler et al. [15], 30 patients with pars defects were assessed on the cause and progression of the disease for over 45 years, and the results revealed that the risk of the progression of spondylolisthesis in patients with bilateral pars defect is similar to that of the general population. Furthermore, it was shown that spondylolisthesis apexes in adolescence and declines in adulthood. Consistent findings have been posited by various studies, providing support for the comparatively benign history of spondylosis, calling for non-operative management [9, 16]. Thus, conservative managements, including physiotherapy with rigid braces, nonsteroidal anti-inflammatory medications, steroidal injections, and avoidance of exacerbating injuries constitute the primary treatment and could be successful for a considerable number of symptomatic patients.

Surgery remains a viable option for patients with unsuccessful of non-operative management. Inherently, the spondylolysis constitutes pseudarthrosis of the isthmus with a failure of the fracture consolidation. The purpose of reconstruction of the pars defect is to strengthen the isthmus, to reinstate the anatomy and the strength of the spine, and maintain the mobility of the concerned level [17]. Thus, solid osteosynthesis with a compression device as well as cancellous bone graft is mandatory.

Ideally, direct pars repair shall be preferred according to the intervertebral disc and facet degenerative changes. Louis et al. [18] suggested that direct repair of the pars defect could be applied to patients either with normal disc or moderate signs of disc degeneration. Grade three of Pfirrmann’s classification corresponds to a mild collapse of the intervertebral disc space with a diffuse loss of signal (moderate sign of disc deterioration) constitutes an ideal limit for direct pars repair technique.

There is no consensus about the optimal and standard direct repair technique for treating pars defect in symptomatic patients. Various surgical techniques with modifications, including Buck’s technique in 1970s, hooks and screws by Morscher in 1984, transverse process wiring technique by Scott in 1986, among others have been documented with various outcomes [3, 19–22]. The Buck technique of direct intra-laminar screw fixation of the defects provided a low-profile implant with restricted dissection. Initially proposed by Buck, the technique was recommended for the fixation of fewer than 3 mm pars defect using Arbeitsgemeinschaft fur Osteosynthesfragen (AO) 4.5-mm stainless steel lag screws [19]. However, the lamina narrowness posed great difficulties for the correct positioning of the screws. It as well lessened the available space for bone grafting, which remains a crucial part of the surgery [23]. Complication rate ranging from 5.6 to 40% was associated with this surgical approach or hardware failure [24]. As illustrated by Scott, placing the wires beneath the transverse processes might be challenging, and perioperative complications, including wire breakage, bleeding, and nerve root injury could occur in 14% of all cases [25]. Therefore, techniques above are unsuccessful in creating adequate compression and stable fixation to the pars defect, which would permit bony ingrowth of the bone graft without fracturing the lamina or the transverse procedure, and without injuring the nerve, or irritating the facets. Then, the laminar hook introduced by Morscher in 1984 allowed fixation of the posterior arch and the bone graft under compression [20]. Despite the healing rates of defect ranging from 56 to 82%, instrumentation failures including screw breakage or implant loosening, were frequently reported, and a comparative biomechanical weakness of the device was described [26–28]. Pedicle screw-rod construct offers more stiffness to flexion loads and large space available for bone grafting, which is supported by various clinical and biomechanical evidence [3, 24, 29, 30]. However, extensive dissection of para-spinal muscles and iatrogenic instability are unavoidable.

Minimally invasive surgeries are evolving, which seek to minimize collateral damage with open procedures. For instance, the use of endoscopy for Buck’s screw placement has been described by Sairyo [31]. Despite of improved clinical outcomes and biomechanical stability of the defect, this demanding technique was challenging to perform on small-bodied patients, for instance, among the Asian population. Moreover, unintended laminar breakage might occur during the screw placement maneuver. Additionally, a new minimally invasive method of repairing pars defects through an illuminated tubular system (22-52 mm in length and 30 degrees of open-angle) was developed. Noteworthy, all direct repair techniques aforementioned including minimally invasive procedures are all performed under general anesthesia. Increasing research evidence constituting animal data, notably, nonhuman primates, reveals that there are adverse effects associated with general anesthetics on the young brain undergoing substantial growth and maturation [32].

Herein, we established a combination of varied minimally invasive techniques to achieve less iatrogenic damage as well as more rapid recuperation of young patients with lumbar spondylolysis. The application of endoscopy and percutaneous screws lessened the amount of disruption on soft tissues to the extent that the surgical intervention was condoned without general anesthesia. Local anesthesia supplemented with epidural anesthesia minimized the adverse effects associated with general anesthesia. It as well permits live neurological surveillance through feedback from patients. Moreover, we established that cleaning the scar and completing the fusion through endoscopy (10 mm in width), which minimized the para-spinal muscle damage. The strength of the hardware (screws and rods) avoided the necessity of postoperative immobilization, and patients participate in routine activities in the first week after the surgery. This procedure must be able to produce a fusion rate equivalent to those of aforementioned techniques. Despite the comparatively short follow-up period in this study, it was adequate to assess the effectiveness of the designed technique to obtain fusion of the pars interarticularis. These component techniques, especially endoscopy, were previously applied in clinical practice with success. However, integrating these two techniques made this study novel and unique. In any circumstances, as little aggressive surgery as possible seems a viable option for young patients with lumbar spondylolysis. However, it’s crucial to recognize, however, that the endoscopic procedure (i.e., refreshment of the defects) might not be indispensable in all cases. When the injury is relatively fresh and narrow, solely mono-segment fixation such as pedicle screw-hook system or percutaneous pedicle screw system could be sufficient enough for the compression of the defect and the occurrence of new fusion ultimately [33].

The present study does have significant limitations. First, the patient selection bias was unavoidable due to its retrospective nature. Second, the endoscopic procedure has a relatively steep learning curve. The cleaning of the pars defect can be done by halving for surgeons who are not specialized in endoscopic techniques. Third, as an initial report, the small sample size makes it difficult to draw definite conclusions as to its efficacy and safety. Therefore, prospective studies with a larger sample size are mandatory for the evaluation of this minimally invasive procedure.

## Conclusion

Herein, we suggest that percutaneous endoscopic-assisted repair of pars defect supplemented with percutaneous pedicle screw fixation could be a reliable minimally invasive treatment alternative fors symptomatic lumbar spondylolysis in the young or highly motivated group of patients.

## Data Availability

The data which analyzed during the study are stored in our hospital and are available from the corresponding author on reasonable request.

## Abbreviation

CT: computed tomography
MRI: magnetic resonance imaging
ODI: Oswestry Disability Index
VAS-B: visual analogue scale for back
VAS-L: visual analogue scale for leg
SF-36: 36-Item Short Form Health Survey
MCS: mental component summary
PCS: physical component summary
LBP: low back pain
DR: direct repair
EA: epidural anesthesia
AO: Arbeitsgemeinschaft fur Osteosynthesfragen
